# Dependence of spin-orbit torque effective fields on magnetization uniformity in Ta/Co/Pt structure

**DOI:** 10.1038/s41598-019-47125-4

**Published:** 2019-07-25

**Authors:** Feilong Luo, Qi Ying Wong, Sihua Li, Funan Tan, Gerard Joseph Lim, Xuan Wang, Wen Siang Lew

**Affiliations:** 1 School of Physical and Mathematical Sciences, Nanyang Technological University, 21 Nanyang Link, Singapore, 637371 Singapore; 2 Department of Physics, School of Science, Lanzhou University of Technology, Lanzhou, 730050 PR China

**Keywords:** Nanoscience and technology, Physics

## Abstract

The spin-orbit torque (SOT) effective fields, namely field-like and damping-like terms, depend on the thicknesses of heavy metal (HM) and ferromagnetic metal (FM) layers, in a stack comprising of HM/FM/HM or oxide. In this work, we report on the dependence of the SOT effective fields on the magnetization uniformity in the wires comprising of Ta/Co/Pt layer structure. SOT dependence on magnetization uniformity dependence was investigated by concurrent variation of the magnetization uniformity in Co layer and characterization of the SOT effective fields in each wire which excludes the layer thickness dependence influences. Our experimental results reveal that the field-like term decreases while the damping-like term increases with increasing Co magnetization uniformity. The magnetization uniformity influence on the effective fields is attributed to the spin Hall effect, which contributes to the SOT.

## Introduction

The control of magnetization switching in magnetic structures by an electric current is crucial for the development of spintronics devices^1^. An established approach is *via* spin-transfer torque (STT) which involves the transfer of angular momentum from spin-polarized current to the magnetization^2^. Lately, current-induced spin-orbit torque (SOT) has emerged as an efficient alternative to STT^3,4^. The SOT is observed in magnetic multilayer structures composed of a ferromagnetic (FM) layer sandwiched by two heavy metal (HM) layers^5–7^. In this structure, the conduction electrons of FM and HMs exhibit strong spin-orbit coupling which leads to two well-known phenomena, *i*.*e*., the Rashba effect and the spin Hall effect (SHE)^3,4^. Due to the Rashba effect, spins accumulate in the FM layer, which exerts both a damping-like and field-like torque on the magnetization of FM layer^8^. Meanwhile, due to SHE, polarized spins are induced to accumulate at the FM/HM interface and diffuse into FM layer, which gives rise to an STT effect on the magnetization^9^. The torque from the Rashba effect and SHE is namely the SOT comprising of a field-like torque and damping-like torque. Generally, the two torques are represented by the two corresponding effective fields, field-like term *H*_F_ and damping-like term *H*_D_^3–7,9,10^.

The dependence of the two effective fields on the orientation of the magnetization has been studied, especially in materials with perpendicular magnetic anisotropy (PMA)^11–17^. Reported SOT measurements on Ta/CoFeB/MgO structures have shown that the damping-like term *H*_D_ changed the direction when the magnetization is reversed^12^. Recently, the dependence of the field-like term *H*_F_ which has been considered as a constant on the polar angle of magnetization was observed experimentally in films with PMA^14,15^. The SOT effective fields also depend on the thicknesses of the FM and HM layers in Ta/CoFeB/MgO structure^18^. The increase of both field-like and damping-like terms with respect to the thickness of Ta has been reported, which is due to a more significant amount of current in a thicker Ta layer. For the dependence on the thickness of the FM layer, the field-like term decreases with increasing CoFeB thickness, while the damping-like term remains constant. Such dependence was ascribed to the giant magnetoresistance effect^18^. In the investigations of the referred dependences, sweeping magnetic fields were used. However, the sweeping behavior, *i*.*e*., magnetizing process, gives rise to magnetization variation or magnetization non-uniformity in magnetic devices. Hence, to precisely characterize the dependencies, the relationship between the SOT effective fields and the magnetic uniformity is required. Specifically, this relationship in a magnetic system with in-plane magnetic anisotropy (IMA) requires investigation, as the SOT devices based on IMA show promising application in spintronics^19^.

Here, we demonstrate the dependence of the SOT effective fields on the magnetization uniformity in wires consisting of Ta/Co/Pt layers with IMA. The impact of HM and FM layers thickness dependence of SOT is eliminated. Varying the uniformity and characterizing the SOT effective fields were achieved concurrently in each wire by applying a magnetic field along the long axis of the wire. Experimental results show that the field-like term decreases with respect to the magnetization uniformity, whereas the damping-like term increases. It is proposed that the magnetization uniformity increase leads to an increase of electron diffusion constant to decrease the field-like term and increase damping-like term.

## Experiments and Discussion

Harmonic Hall resistance measurement technique, which has been reported earlier^20^, were employed to characterize the SOT effective fields. In this technique, a constant field, *H*_*x*−ext_, is applied longitudinally to the wire long axis to ensure a constant magnetization uniformity, while a transverse field to the wire, *H*_*y*−ext_, is swept in the plane to obtain the SOT effective fields accurately. The transverse field changes the magnetization azimuthal angle *φ*_0_. With *H*_*x*−ext_ being constant, the cosine of the angle, *X*, which can be simultaneously calculated by $X=\,\cos \,{\phi }_{0}={H}_{x-{\rm{ext}}}/\sqrt{{H}_{x-{\rm{ext}}}^{2}+{H}_{y-{\rm{ext}}}^{2}}$. *X* is used to apply in the expression of the second harmonic Hall resistance *R*_2ndHall_ which is:

1 $${R}_{{\rm{2nd}}{\rm{Hall}}}=\frac{{R}_{{\rm{AHE}}}}{2{H}_{\perp }}{H}_{{\rm{D}}}X+\frac{{R}_{{\rm{PHE}}}}{{H}_{x-{\rm{ext}}}}{H}_{{\rm{F}}}(2{X}^{4}-{X}^{2}),$$

where *H*_*D*_ and *H*_*F*_ are the damping-like term and field-like term, respectively, *R*_AHE_ and *R*_PHE_ are the amplitudes of anomalous Hall effect and planar Hall effect resistances, and *H*_⊥_ is the effective field orientating the magnetization in the film plane. By fitting the experimental second harmonic Hall resistance with Eq. (1), the SOT effective fields can be extracted. In Eq. (1), the parameter *R*_PHE_/*H*_*x*−ext_ can be obtained from the first harmonic Hall resistance *R*_1st Hall_, which is expressed as ${R}_{1stHall}={R}_{PHE}\,\sin \,2{\phi }_{0}$. Due to the expression of $\cos \,{\phi }_{0}$, the maximum and minimum values of *R*_1st Hall_ occur at *H*_*y*−ext_ = ±*H*_*x*−ext_, which give *R*_PHE_.

**Figure 1:**
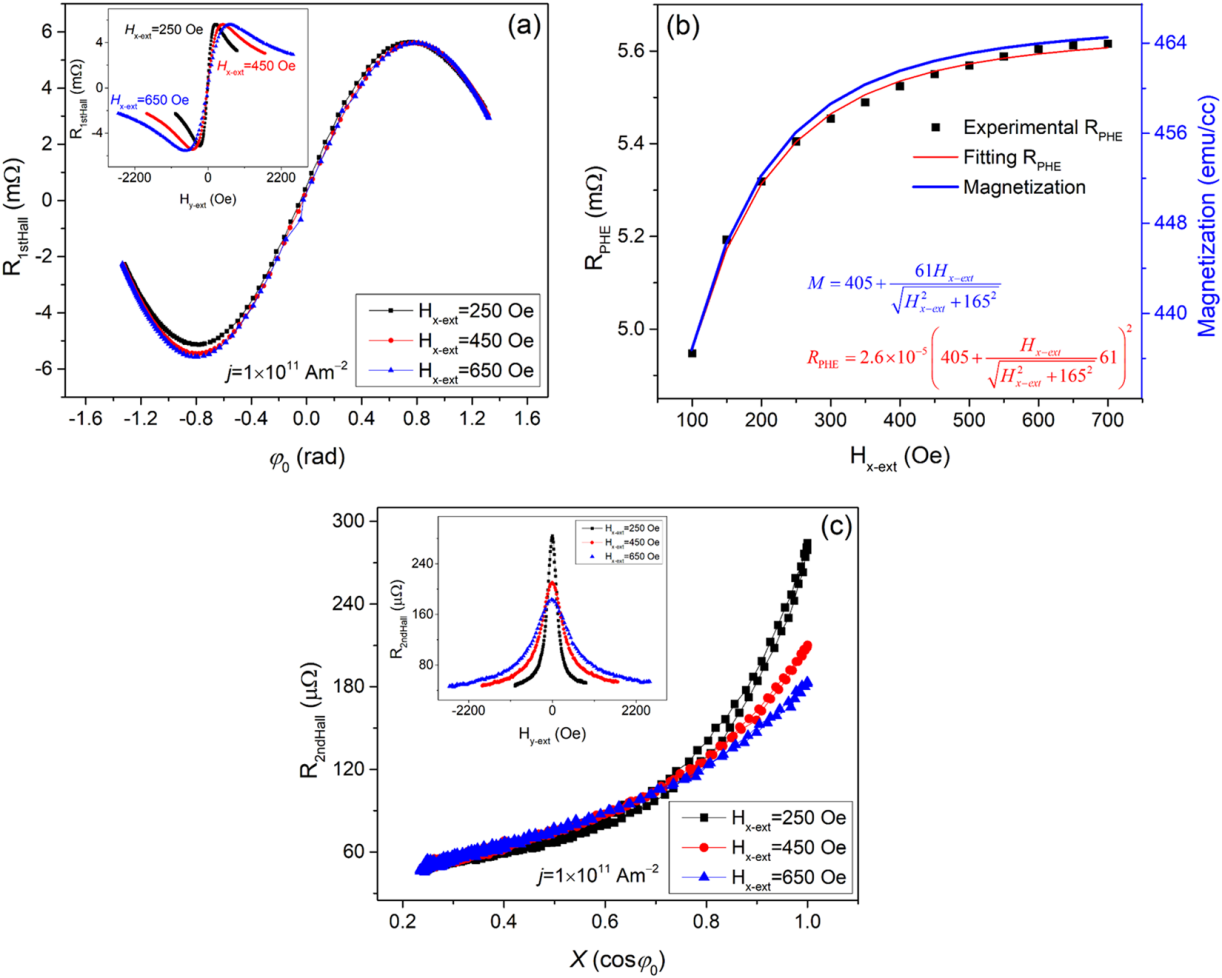
(**a**) The measured first harmonic resistances *R*_1st Hall_ with respect to the azimuthal angle *φ*_0_ of magnetization. Inset is the measured *R*_1st Hall_ with respect to the applied transverse field *H*_*y*−ext_. (**b**) The measured *R*_PHE_ and the calculated *M*, with respect to the longitudinal field. *R*_PHE_ is obtained by fitting the sin2*φ*_0_ curves of (**a**). (**c**) The measured second harmonic Hall resistances *R*_2ndHall_ with respect to the cosine *X* of the azimuthal angle. Inset is the measured *R*_2ndHall_ with respect to applied transverse field *H*_*y*−ext_.

The measurements were carried out in the wires with stacks of Ta(*t* nm)/Co(2 nm)/Pt(5 nm), where *t* = 4, 6, 8 and 10^20^. The fabrication and patterning processes of the wires are described elsewhere^17,20–22^. The SOT fields are quantified as a function of the longitudinal fields in the sample of Ta(4 nm)/Co(2 nm)/Pt(5 nm)^20^. In this quantification, the constant longitudinal field *H*_*x*−ext_ was applied in a range of 250 Oe to 650 Oe with a 50 Oe increment. For each value of *H*_*x*−ext_, the ratio of the maximum value of the sweeping field *H*_*y*−ext_ to *H*_*x*−ext_ was fixed. An AC frequency of 307.1 Hz was used for the low-frequency harmonic Hall resistance measurements. The amplitudes of the AC current were in the range of 3 × 10^10^~10 × 10^10^ Am^−2^ with an increment of 10^10^ Am^−2^. The first and second harmonic Hall resistances were measured using a 7265 DSP lock-in amplifier. The obtained harmonic Hall resistances at applied fields *H*_*x*−ext_ = 250 Oe, 450 Oe, and 650 Oe and current density of 1 × 10^11^ Am^−2^ are shown in Fig. 1.

The measured first harmonic Hall resistances *R*_1st Hall_ exhibit typical $\sin \,2{\phi }_{0}$ behaviors as functions of the azimuthal angle *φ*_0_ of the magnetization, and the minimum and maximum values of *R*_1st Hall_ are at *φ*_0_ = ±45 degrees. Correspondingly, for each value of *H*_*x*−ext_ shown in the inset of Fig. 1(a), the minimum and maximum values of *R*_1st Hall_ occur at *H*_*x*−ext_ = ±*H*_*y*−ext_, which give the values of *R*_PHE_ and the ratio of *R*_PHE_/*H*_*x*−ext_ shown in Fig. 1(b). In Fig. 1(c), the measured second harmonic Hall resistances, *R*_2nd Hall_, are shown to increase with increasing *X* for each value of *H*_*x*−ext_. Fitting the experimental *R*_2nd Hall_ by Eq. (1), where the values of *R*_PHE_ and *R*_PHE_/*H*_*x*−ext_ are recorded in Fig. 1(b), we compute the two effective SOT fields, *H*_F_ and *H*_D_.

**Figure 2:**
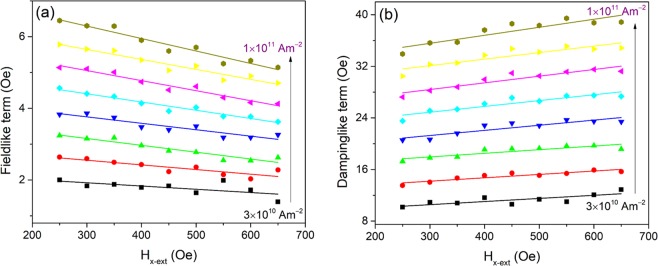
The measured field-like term (**a**), and damping-like term (**b**), with respect to the longitudinal field.

As shown in Fig. 2, for each value of *H*_*x*−ext_, *H*_F_ and *H*_D_ increase with respect to the current density at each value of *H*_*x*−ext_. The values of *H*_F_ and *H*_D_ are similar to that reported in the same stack^20^. We notice that *H*_F_ and *H*_D_ vary with the longitudinal field for each value of the applied current densities. However, at the current density of 3 × 10^10^ Am^─2^, the field-like term variation is 19.7%, and the damping-like term variation is 16.9% when *H*_*x*−ext_ increases from 250 Oe to 650 Oe. At the current density of 10^11^ Am^─2^, the field-like term variation increases to 21.5% and the damping-like term variation decrease to 12.4%. The variations are not only the functions of the magnetization uniformity but also the applied current. The role of the current in the variation indicates that possible thermal effect, such as the anomalous Nernst effect, should exist during the SOT measurement^23–26^. In the Ta/Co/Pt stack where our measurements were carried out, there are differences in the resistivity of Ta and Pt. Due to Joule heating which is induced by electric current, the different resistances give rise to a thermal gradient along the normal direction of the stack. The thermal gradient contributes to the measured second harmonic Hall resistances which were used to calculate the SOT effective fields^13^. Depending on the direction of the thermal gradient; the thermal contribution may lead to an over or underestimation of the SOT effective field when the current density increases. However, in our measurement regime, the thermal effects are considered negligible because the current density in our experiments was in the range of ~10^7^ A/m^2^, where Joule heating is generally negligible^5^. Furthermore, both the damping like term and field like term relates monotonically with the applied current density as shown in Fig. 2, implying that the thermal effects insignificantly to the SOT effective field^27,28^.

The magnetization uniformity is equivalent to magnetization amplitude M for the magnetic wire. This magnetization amplitude can be characterized with respect to the applied longitudinal field by measuring *R*_1st Hall_, thereby obtaining the *R*_PHE_. As shown in Fig. 1(b), *R*_PHE_ increases with respect to *H*_*x*−ext_, where *H*_*x*−ext_ is extended to 100 Oe. This increase is attributed to the polycrystalline structure of the Co layer in the sputtered Ta/Co/Pt film. Without applying *H*_*x*−ext_, the magnetic moment of Co crystalline grains orientates randomly in the film. This is due to the random orientation of the effective field *H*_crys_ generated by the crystalline magnetic anisotropy of each grain. Hence, the *M* of the wire equals the value of remanence magnetization *M*_r_, which is determined by the intrinsic demagnetizing field transverse to the wire.

**Figure 3:**
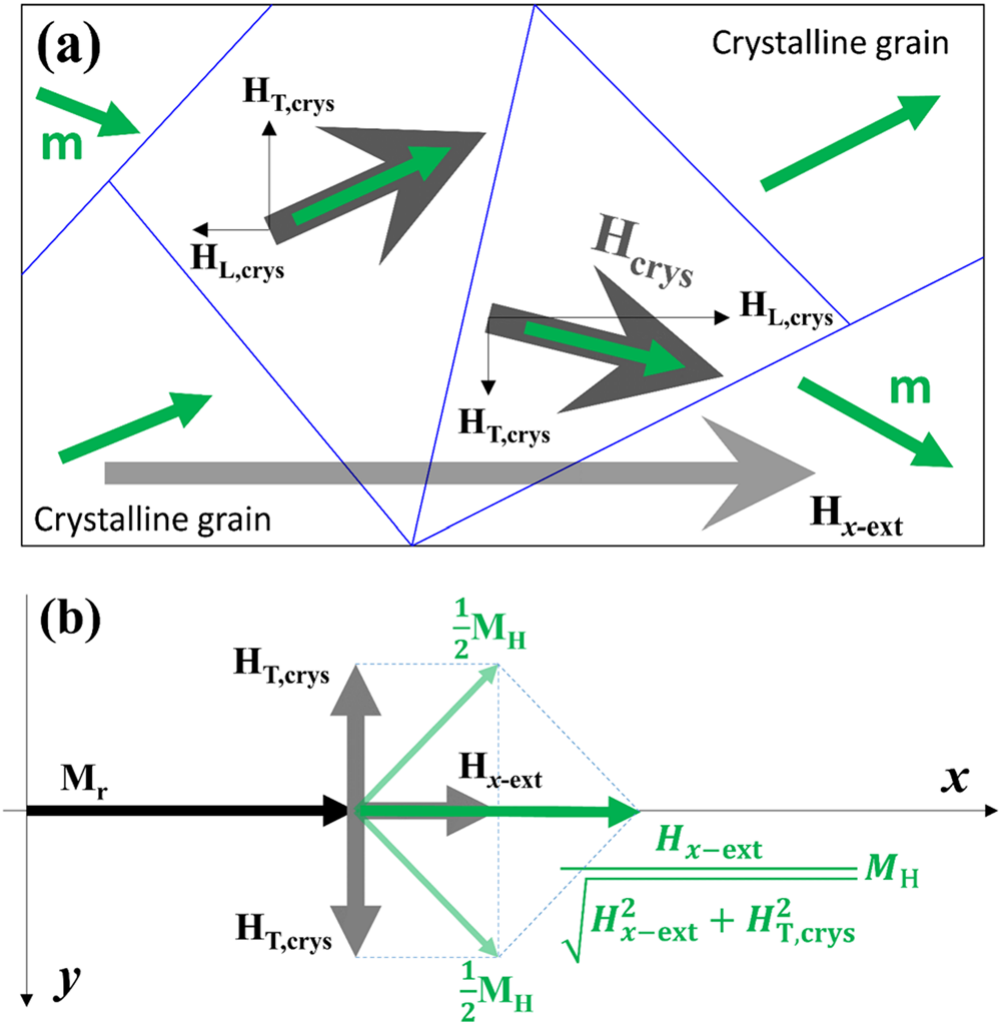
(**a**) The schematic of a polycrystalline magnetic structure and the orientation of magnetic moment for each of crystalline grain under *H*_*x*−ext_ and transverse demagnetizing field. (**b**) The schematic of the magnetization composition for the magnetic structure.

However, when *H*_*x*−ext_ is applied, the magnetic moment **m** of the grains re-orientates towards the *x*-axis, as schematically shown in Fig. 3(a). Consequently, *M* increases starting from *M*_*r*_. The increase of *M* leads to the increase of *R*_PHE_, as *M* is related to *R*_PHE_ by the expression *R*_PHE_ = *kM*^2^ ^29–31^, where *k* is a material related coefficient. The maximum of *M* is the saturation magnetization *M*_s_. Therefore, *M*_H_, defined as *M*_H_ = *M*_s_−*M*_r_, is the maximum of the magnetization component, which can be manipulated by the external field *H*_*x*−ext_. We consider the magnetization component as the resultant of two vectors expressed by *M*_H_/2, instead of evaluating the contribution from each magnetic grain to the magnetization component. As shown in Fig. 3(b), *H*_*x*−ext_ orientates both vectors along the *x*-axis, while the nonzero *y* component *H*_T,crys_ of *H*_crys_ orientates each vector along the ±*y* direction, respectively. Hence, each *M*_H_/2 orientates at their balanced direction determined by *H*_*x*−ext_ and *H*_T,crys_, as shown in Fig. 3(b). The *y* components of the two vectors cancel each other, while the *x* component of each vector contributes to the magnetization component as $({H}_{x-ext}/\sqrt{{H}_{x-ext}^{2}+{H}_{T,crys}^{2}})({M}_{H}/2)$. Consequently, the expression of the magnetization component is obtained as $({H}_{x-ext}/\sqrt{{H}_{x-ext}^{2}+{H}_{T,crys}^{2}})({M}_{H}/2)$. Therefore, the total magnetization *M* can be expressed as $M={M}_{R}+({H}_{x-ext}/\sqrt{{H}_{x-ext}^{2}+{H}_{T,crys}^{2}}){M}_{H}$, and *M*_r_ + *M*_H_ equals to the saturation magnetization *M*_s_ of the wires. Considering *M*_r_ + *M*_H_ = 466 emu/cc for the sample of Ta (4 nm)/Co (2 nm)/Pt (5 nm)^20^, and substituting the *M* expression into the *R*_PHE_ expression, we fit the measured *R*_PHE_ as shown in Fig. 1(b). The fitting plot of *R*_PHE_ matches the experimental *R*_PHE_, which verifies the analytical expression of *M* and gives $M=405+({H}_{x-ext}/\sqrt{{H}_{x-ext}^{2}+{165}^{2}})61$ emu/cc.

**Figure 4:**
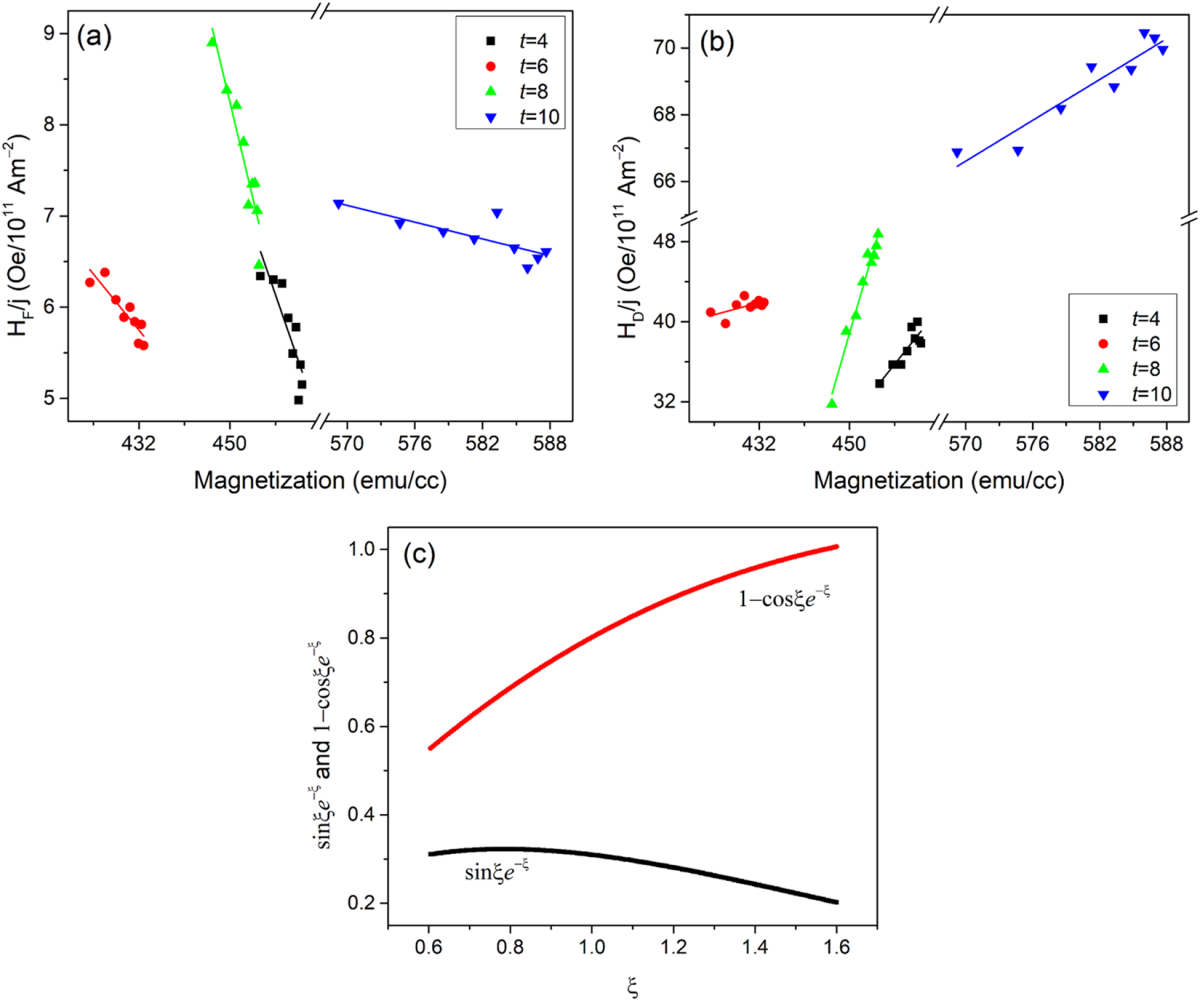
The measured field-like term per current density (**a**), and damping-like term per current density (**b**), with respect to the calculated magnetization *M* for Ta(*t* nm)/Co(2 nm)/Pt(5 nm), where *t* = 4, 6, 8 and 10. (**c**) The plots of sin*ξe*^−*ξ*^ and 1−cos*ξe*^−*ξ*^ with respect to *ξ*.

The SOT effective fields per 10^11^ Am^−2^ at each value of *H*_*x*−ext_ were obtained from Fig. 2, for comparison. Replacing *H*_*x*−ext_ with the corresponding value of *M* shown in Fig. 1(b), the SOT effective fields per 10^11^ Am^−2^ with respect to *M* are plotted for sample Ta (4 nm)/Co (2 nm)/Pt (5 nm) in Fig. 4. Similarly, the SOT effective fields per 10^11^ Am^−2^ and the magnetization were quantified for samples Ta (*t* nm)/Co (2 nm)/Pt (5 nm), where *t* = 6, 8 and 10. As reported previously^20^, similar saturation magnetization values for the samples *t*_Ta_ = 4 nm and *t*_Ta_ = 8 nm leads to similar tendencies of the field like and damping like fields due to their dependence on magnetization. While substantial differences in the saturation magnetization values for samples *t*_Ta_ = 6 nm and *t*_Ta_ = 8 nm leads to different tendencies of the field-like and damping like SOT fields. As shown in Fig. 4(a,b), the field-like term decreases with respect to the magnetization magnitude while the damping-like term increases in each sample.

We demonstrate that the dependence of the SOT effective fields on the magnetization magnitude or uniformity is attributed to SHE in the Ta/Co/Pt structure. SHE-induced spin accumulation, **s**, which is at the interfaces of Ta/Co and Co/Pt, diffuses into the FM layer to cause STT on the magnetization^9^. In the STT model proposed by S. Zhang^32^, the spin current, which is from a reference layer, leads to transverse spin accumulation in the free layer. Consequently, the transverse spin accumulation induces two effective fields: *b***m**_*r*_ and *a***m**_*f*_ × **m**_*r*_, where **m**_*r*_ and **m**_*f*_ are unit vectors of the local magnetization of the reference layer and the free layer, respectively. When **m**_*r*_ and **m**_*f*_ are in the planes of the magnetic layers, *b* and *a* are expressed as $b=(h{j}_{e}/e{M}_{s}{t}_{F})\sin \,\xi .{e}^{-\xi }$ and $a=(h{j}_{e}/e{M}_{s}{t}_{F})(1-\,\cos \,\xi .{e}^{-\xi })$, respectively, where *h* is the Planck constant, *j*_*e*_ is the electric current density perpendicular to the plane of magnetic layers, *t*_F_ is the thickness of the free layer, and *e* is the electron charge. In the expressions of *a* and *b*, *ξ* equals to ${t}_{F}/\sqrt{2}\lambda$ with a spin diffusion length ${\lambda }_{f}=\sqrt{2h{D}_{0}/J}$, where *J* is a coefficient of the contact interaction between the spin accumulation and the local magnetization of the free layer, and *D*_0_ is the electron diffusion constant. Analogously in the Ta/Co/Pt structure, the Ta or Pt layer is used to generate spin current normal to the magnetic Co layer. Hence, the Ta or Pt layer is similar to the reference layer, as such, **s** can be considered as **m**_*r*_. The spins generated by the Ta and Pt layers are accumulated at the Co layer, which allows us to take the Co layer as analogous to the free layer. Similarly, **m** is to **m**_*f*_. Consequently, the field-like term, *H*_F_ is equivalent to *b****m***_*r*_, and the damping-like term, *H*_D_ is equivalent to *a***m**_*f*_ × **m**_*r*_. Thus, we obtain ${H}_{F}=(h{j}_{e}/e{M}_{s}{t}_{f})(\sin \,\xi .{e}^{-\xi })$ and ${H}_{D}=(h{J}_{e}/e{M}_{s}{t}_{F})(1-\,\cos \,\xi .\,{e}^{-\xi })$ for the Ta/Co/Pt samples, where *J*_e_ is the charge current.

We propose that the magnetization magnitude of Co layer manipulate the SOT effective fields *via* the coefficient *ξ*, considering the above expressions of *H*_*F*_ and *H*_*D*_. The damping-like term is related to spin Hall angle *θ*_SH_ *via* the expression *H*_*D*_ = *θ*_*SH*_*hJ*_*e*_/*eM*_*s*_*t*_*F*_, where *θ*_SH_ is defined as the ratio of spin current *j*_s_ to charge current *j*_e_^19,33–35^. Comparing the two expressions of *H*_D_, we obtain ${\theta }_{SH}=1-\,\cos \,\xi {e}^{-\xi }$ As such, *ξ* is ≤ 1.6, since the sum of Pt and Ta spin Hall angles is ≤1^7,36^. As $\xi ={t}_{F}/(\sqrt{2}{\lambda }_{J})$ and *λ*_*J*_ is about 1.2~2.4 nm for Co^25^, we obtain *ξ* ≥ 0.6, using the Co layer thickness *t*_F_ = 2 nm. Therefore, our samples have values of 0.6 ≤ *ξ* ≤ 1.6. *ξ* can be rewritten as $\xi =({t}_{F}/2)\sqrt{J/h{D}_{0}}$, where *D*_0_ is related to the magnetization of the wire^30,37^. Ustinov created a superlattice model to explain the correlation of *D*_0_-related magnetoresistance (MR) and magnetization^37^. In this model, the superlattice comprises of several magnetic layers, and for any of two adjacent layers, magnetizations are initially antiparallel to each other. A transverse magnetic field, which is perpendicular to the initial magnetization in the plane of the magnetic layers, is applied to change the magnetization amplitude of the superlattice. The model concludes that the MR increases with respect to the magnetization of the superlattice. Hence, *D*_0_ decreases with increasing magnetization in our samples, as it is inverse proportional to MR. Therefore, *ξ* increases with respect to the magnetization magnitude, due to $\xi =({t}_{F}/2)(/)(\sqrt{J/h{D}_{0}})$. In the range of 0.6~1.6 for our samples, the increase of *ξ* leads to the decrease of the term $\sin \,\xi {e}^{-\xi }$ and increase of the term 1−cos*ξe*^−*ξ*^, as shown in Fig. 4(c). Therefore, *H*_F_ decreases and *H*_D_ increases with respect to the magnetization, respectively, as the term *hj*_*e*_/*eM*_*s*_*t*_*F*_ is a constant for each sample.

## Conclusion

In conclusion, our measurement results show that the SOT effective fields depend on the magnetization uniformity in Ta/Co/Pt structure. The dependence indicates that the SOT effective fields can be manipulated by varying the magnetization uniformity. The change of magnetization uniformity was achieved in each sample by applying magnetic fields along the long axis of the wire. As the SOT effective fields are concurrently characterized, our characterization method eliminates influences from other SOT dependence effects. As an analogy to the STT effect from a reference layer, the SOT dependence on the magnetization uniformity is attributed to the electron diffusion properties. This dependence suggests that SHE plays a significant role in the dependence of SOT effective fields on magnetization. It also indicates that the SOT effective fields cannot be considered as constant parameters when analyzing domain wall dynamics *via* SOT. Moreover, we conclude that magnetization enhances the damping-like torque while suppressing the field-like torque.
